# Heterozygous *Mylk3* Knockout Mice Partially Recapitulate Human DCM With Heterozygous *MYLK3* Mutations

**DOI:** 10.3389/fphys.2019.00696

**Published:** 2019-06-06

**Authors:** Carson L. Tougas, Tabor Grindrod, Lawrence X. Cai, Fariz F. Alkassis, Hideko Kasahara

**Affiliations:** Department of Physiology and Functional Genomics, College of Medicine, University of Florida, Gainesville, FL, United States

**Keywords:** kinase, heart, genetic dilated cardiomyopathy, heterozygous knockout, animal model of human disease

## Abstract

**Backgrounds:** Recent studies identified heterozygous variants in *MYLK3* gene that encodes cardiac myosin light chain kinase (cMLCK) are related to familial dilated cardiomyopathy (DCM) for the first time. Autosomal dominant traits suggest that pathogenesis of DCM could be related to heterozygous *MYLK3* loss-of-function variants (haploinsufficiency). We previously generated and examined homozygous *Mylk3* knockout mice that lead to heart failure. It had yet to be examined whether heterozygous *Mylk3* knockout mice represent a DCM-like phenotype.

**Methods and Results:** Heterozygous knockout (*Mylk3*^wild/-^) mice were examined regarding cardiac function, heart histology and expression of cMLCK protein and mRNA relative to age-matched wild-type controls (*Mylk3*^wild/wild^). At 4 months of age, cardiac contractility in heterozygous knockout mice was reduced with percent fractional shortening of 23.3 ± 1.2% compared to 30.1 ± 1.8% in control (*Mylk3*^wild/-^ vs. *Mylk3*^wild/wild^, *n* = 9 each). In 4-month-old heterozygous knockout hearts, expression of cMLCK mRNA was expectedly reduced by almost half, however, protein expression was reduced by approximately 75% relative to the control wild-type (*Mylk3*^wild/-^ vs. *Mylk3*^wild/wild^, *n* = 9 each). Isolated ventricular cardiomyocytes from heterozygous knockout mice were larger with increase of short-axis length relative to the cardiomyocytes from control mice. However, increase of heart failure markers as well as interstitial fibrosis were not evident in heterozygous knockout mice compared to controls.

**Conclusion:** Heterozygous *Mylk3* knockout mice show mild reduction of cardiac contractility by 4 months of age, and proteins reduced by approximately 75% relative to the control wild-type mice. These mice partly resemble human with the heterozygous *MYLK3* mutation, but the reduction in cardiac contractility was milder.

## Introduction

The dynamics of cardiac contraction and relaxation are fundamentally related to actin-myosin interactions. Myosin is a hexamer composed of two heavy chains and two pairs of light chains (MLC1 and MLC2). Ventricular MLC2 (MLC2v) is bound to the neck of the heavy chain, which acts as a lever arm to generate movement, and MLC2v phosphorylation has been shown to potentiate the rate andVerify that all the equations and special characters are displayed correctly. force of cardiac contraction (Sanbe et al., 1999; Davis et al., 2001; Moss and Fitzsimons, 2006; Stelzer et al., 2006; Scruggs and Solaro, 2011). The predominant kinase for MLC2v is cardiac MLCK (cMLCK) encoded by *Mylk3* in mice and *MYLK3* in humans. Reduced phosphorylation of MLC2v has been implicated in human heart disease where phosphorylation was reduced to ∼18% of total MLC2v in failing hearts from ∼30 to 40% in healthy hearts (Morano, 1999; van der Velden et al., 2003). In addition, recent studies showed that heterozygous human mutations in *MYLK3* gene could be related to familial dilated cardiomyopathy (DCM) (Tobita et al., 2017; Hodatsu et al., 2019).

Haploinsufficiency due to loss-of-function mutations has been documented as a pathological mechanism for a number of human diseases (Marston et al., 2012; Rocha et al., 2016; Bartha et al., 2018). The autosomal dominant nature of *MYLK3*-related DCM suggests that heterozygous loss-of-function may be responsible for pathogenesis. Indeed, homozygous *Mylk3* knockout (*Mylk3*^-/-^) hearts were moderately enlarged with increased heart weight at 3 and 6 months of age in sedentary conditions relative to age- and sex-matched (female) control wild-type litters. MLC2v phosphorylation was below the level of detection. Cardiac contraction assessed using magnetic resonance imaging and echocardiography demonstrated a reduction in ejection fraction and increases in the volume of the left ventricular cavity both at end-systole and end-diastole. However, these mice did not show typical heart failure phenotypes, such as an increase in interstitial fibrosis or fetal gene re-expression (Warren et al., 2012). Isolated ventricular cardiomyocytes from adult *Mylk3*^-/-^ mice were larger than cardiomyocytes from control mice and displayed reductions in contractility and speed of relaxation with no changes in amplitude of the intracellular Ca^2+^ transient. In contrast to a moderate reduction in contractility under basal conditions, 3 months of transverse aortic constriction resulted in profound heart failure and a decreased survival rate in these mice.

Heterozygous *Mylk3* knockout (*Mylk3*^wild/-^) knockout mice remain unexplored as a human DCM model. The aim of this study is to assess the phenotypic similarities and differences between heterozygous *MYLK3* variants in DCM patients versus heterozygous *Mylk3* knockout mice. Heterozygous *Mylk3* knockout mice demonstrate mild heart failure with markedly reduced expression of cMLCK proteins discordant with mRNA level.

## Materials and Methods

### Mouse Model

Germline *Mylk3*^wild/-^ mice were generated as reported in our previous study (Warren et al., 2012), followed by backcrossing with C57BL/6 wild-type mice over six generations. Age- and sex-matched (female) wild-type mice were used as controls. All animal experiments were performed with approval from the University of Florida Institutional Animal Care and Use Committee, which conforms to NIH guidelines.

### Echocardiogram and Left Ventricular Pressure Volume (LVPV) Measurement

Echocardiography of the hearts was performed as described previously (Warren et al., 2012). Briefly, mice were anesthetized with 1.5 to 2% isoflurane to maintain heart rate around 500 bpm during imaging using Vevo770 (Visual Sonics). LVPV measurements using a PVR1045 catheter (Millar, Houston, TX, United States) were performed under intubation and ventilation with 1 to 2% isoflurane to maintain heart rate around 450 bpm using standard methods (Pacher et al., 2008) and were analyzed by Lab Chart 8 (AD Instruments, Colorado Springs, CO) followed by the conversion of relative volume unit (RVU) to units of volume using the cuvette calibration.

### Measurements of Cardiomyocyte Cell Size, Simultaneous Measurements of Cell Shortening and Intracellular Free Calcium

Isolated adult cardiomyocytes attached to glass coverslips were imaged under a microscope and digitized for measurements of cell surface area. Rod-shaped cardiomyocytes with clear cross-striations, staircase ends and surface membranes free from blebs were utilized for simultaneous measurements of cell shortening and intracellular free calcium (IonOptix, Westwood, MA, United States), performed as described previously (Takeda et al., 2009).

### Western Blotting, Immunostaining and Histological Analyses

Paraffin-embedded tissue sectioning of 5 μm thickness was used for Picro Sirius Red staining and immunostaining as described previously (Warren et al., 2012). Western blot analyses and immunostaining were performed with the following antibodies: cMLCK (Chan et al., 2008), phospho-MLC2v (gift from Dr. N. Epstein, NIH) (Davis et al., 2001), MLC2 (F109.3E1, ALX-BC-1150-S-L005, Enzo Life Science, Farmingdale, NY, United States), α-actinin2 (mouse monoclonal A7811 Sigma), myomesin (mouse monoclonal, mMAC myomesin B4, Developmental Studies Hybridoma Bank), and GAPDH (MAB374, MilliporeSigma, Burlington, MA, United States). Fluorescent staining was performed side by side, followed by imaging with the same exposure time below the level of saturation using a ZEISS Axiovert200M (Carl Zeiss AG, Oberkochen, Germany).

### Administration of Proteasome Inhibitors

A combination of Lactacystin (10 μM) and MG132 (5 μM) (LifeSensors, Malvern, PA, United States) was added on the day of isolation and incubated for 48 h. Cardiomyocytes were scraped, and used for Western blotting.

### Real-Time RT-PCR

Real-time RT-PCR was performed using TaqMan Gene Expression Assays (Applied Biosystems, Foster City, CA, United States): cardiac MLCK Mm00615292, atrial natriuretic factor (ANF) Mm01255748, and brain natriuretic peptide (BNP) Mm00435304, followed by normalization to β-actin expression (No. 4352933E). Duplicated experiments were averaged.

### Statistical Analyses

Data presented are expressed as mean values ± S.E.M. Results were compared using a student’s *T*-test or ANOVA followed by Fisher’s *post hoc* test (SPSS ver. 25). *P* < 0.05 was considered significant.

## Results

### Heart Enlargement and Reduction of Contractility in Heterozygous *Mylk3* Knockout Mice

**FIGURE 1:**
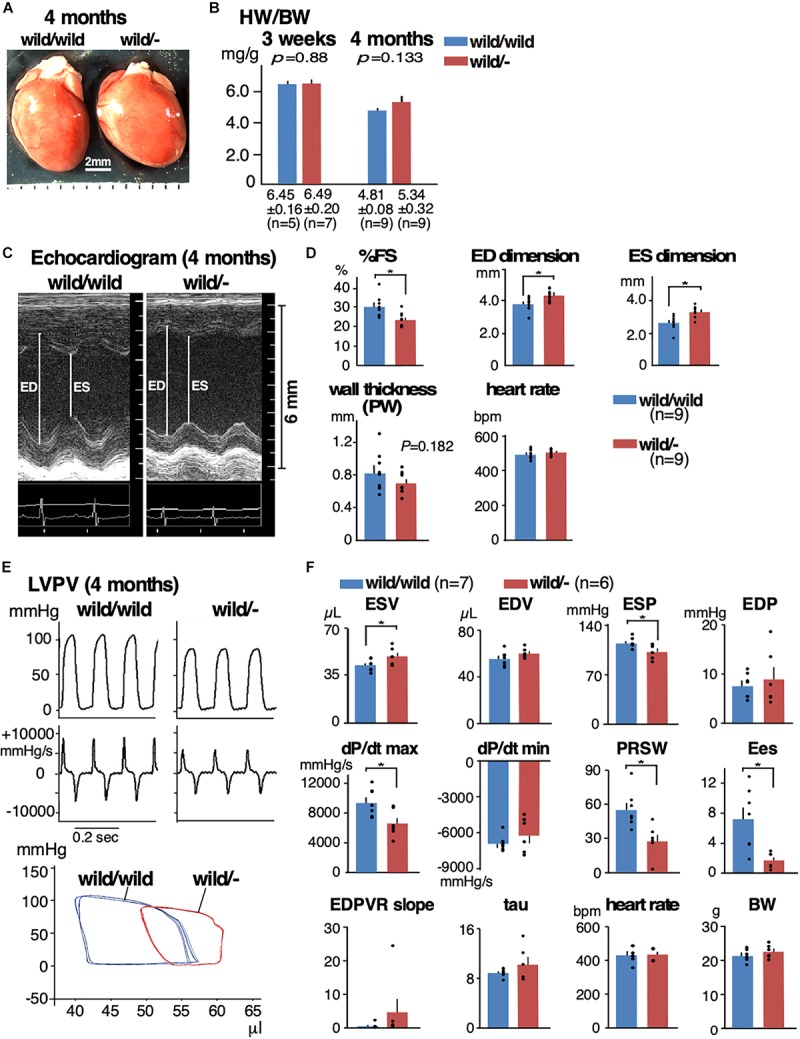
Mild heart enlargement and reduced contractile function in heterozygous *Mylk3* knockout mice. **(A)** By mating *Mylk3*^wild/-^ mice, *Mylk3*^wild/wild^, *Mylk3*^wild/-^, and *Mylk3*^-/-^ were generated. Representative hearts dissected from three groups of mice at 4 months of age. Bar = 2 mm. **(B)** HW/BW (mg/g) at 3 weeks and 4 months of age. **(C,D)** Representative images of M-mode ultrasound and echocardiographic indices of *Mylk3*^wild/wild^ and *Mylk3*^wild/-^ mice at 4 months of age. Representative tracing of LVPV measurements **(E)** and summarized data **(F)** from *Mylk3*^wild/wild^ and *Mylk3*^wild/-^ mice at 4 months of age. Data are expressed as mean ± SEM, with or without individual data that are presented by black circles. *P* values are indicated in panel B, or ^∗^ indicates *P* < 0.05. HW/BW, heart weight/body weight ratio; ED, end-diastolic; ES, end-systolic; %FS, % left ventricular fractional shortening; PW, posterior wall; ESV, end-systolic volume; EDV, end-diastolic volume; ESP, end-systolic pressure; EDP, end-diastolic pressure; dP/dt max, peak rate of pressure rise; dP/dt min, peak rate of pressure decline; PRSW, preload recruitable stroke work; Ees, end-systolic elastance; EDPVR slope, end-diastolic PV relation slope; Tau, relaxation time constant.

By mating heterozygous *Mylk3* knockout mice (*Mylk3*^wild/-^), we generated and analyzed age- and sex-matched (female) *Mylk3*^wild/-^ relative to the control wild-type litters (*Mylk3*^wild/wild^) (**Figure 1A**). There was no change in the heart weight/body weight (HW/BW) ratio between the two groups at 3 weeks of age (6.45 ± 0.16 in control vs. 6.49 ± 0.20 mg/g in heterozygous knockout mice, *n* = 5 and 7, respectively, *P* = 0.88). At 4 months of age, while not significant (*P* = 0.133), HW/BW was increased in heterozygous knockout relative to control mice (4.81 ± 0.08 vs. 5.34 ± 0.32 mg/g, *n* = 9 each) (**Figure 1B**). Hereafter, we examined 4-month-old mice.

Cardiac contractility examined using echocardiogram demonstrated a significant reduction in contractility and an increase in the LV chamber dimensions both at diastole and systole in heterozygous knockout relative to the controls (%fractional shortening, 30.1 ± 1.8 in control vs. 23.3 ± 1.2% in heterozygous knockout mice; end-diastolic dimension, 3.76 ± 0.14 vs. 4.30 ± 0.12 mm; end-systolic dimension, 2.65 ± 0.15 vs. 3.30 ± 0.12 mm, *n* = 9 each, respectively, *P* < 0.05; **Figure 1C,D** and **Table 1**). Hemodynamic measurements also showed dilation of the LV, in particular, at end-systole, a decrease in end-systolic pressure, and a decrease in LV contractility (a decrease of preload recruitable stroke work, PRSW and end-systolic elastance, Ees) in heterozygous knockout relative to the age- and sex-matched control wild-type mice (**Figure 1E,F** and **Table 2**). However, diastolic function was not significantly impaired as represented by the end-diastolic pressure volume relation slope and relaxation time constant (tau).

**Table 1:** Summary of echocardiographic indices of *Mylk3*^wild/wild^ and *Mylk3*^wild/-^ mice at 4 months of age.

	WW (*n* = 9)	W/– (*n* = 9)	*P value*
%FS	30.1 ± 1.8	23.3 ± 1.2	0.006*
ED dimension (mm)	3.76 ± 0.14	4.30 ± 0.12	0.009*
ES dimension (mm)	2.65 ± 0.15	3.30 ± 0.12	0.003*
Wall thickness (PW)	0.83 ± 0.07	0.70 ± 0.04	0.182
Heart rate (bpm)	494 ± 9	503 ± 6	0.417


ED, end-diastolic; ES, end-systolic; %FS, % left ventricular fractional shortening; PW, posterior wall. P, P value. *P < 0.05.

**Table 2:** Summary of echocardiographic indices of *Mylk3*^wild/wild^ and *Mylk3*^wild/-^ mice at 4 months of age.

	WW (*n* = 7)	W/– (*n* = 6)	*P value*
ESV (μL)	42.9 ± 1.7	49.7 ± 2.6	0.044*
EDV (μL)	55.1 ± 2.4	59.8 ± 1.7	0.15
ESP (mmHg)	114 ± 3	102 ± 4	0.031*
EDP (mmHg)	7.5 ± 1.0	9.1 ± 2.3	0.566
dP/dt max (mmHg/s)	9377 ± 707	6532 ± 759	0.019*
dP/dt min (mmHg/s)	-6932 ± 243	-6197 ± 600	0.296
PRSW	54.3 ± 6.1	30.3 ± 6.4	0.020*
Ees	6.96 ± 1.64	1.81 ± 0.59	0.020*
EDPVR slope	0.41 ± 0.29	4.49 ± 3.97	0.352
Tau (ms)	8.87 ± 0.25	10.27 ± 1.10	0.268
Heart rate (bpm)	435 ± 17	433 ± 16	0.948
Body weight	21.2 ± 1.8	22.4 ± 1.8	0.272


ESV, end-systolic volume; EDV, end-diastolic volume; ESP, end-systolic pressure; EDP, end-diastolic pressure; dP/dt max, peak rate of pressure rise; dP/dt min, peak rate of pressure decline; PRSW, preload recruited stroke work; Ees, end-systolic elastance; EDPVR slope, end-diastolic PV relation slope; Tau, relaxation time constant. P, P value. *P < 0.05.

**FIGURE 2:**
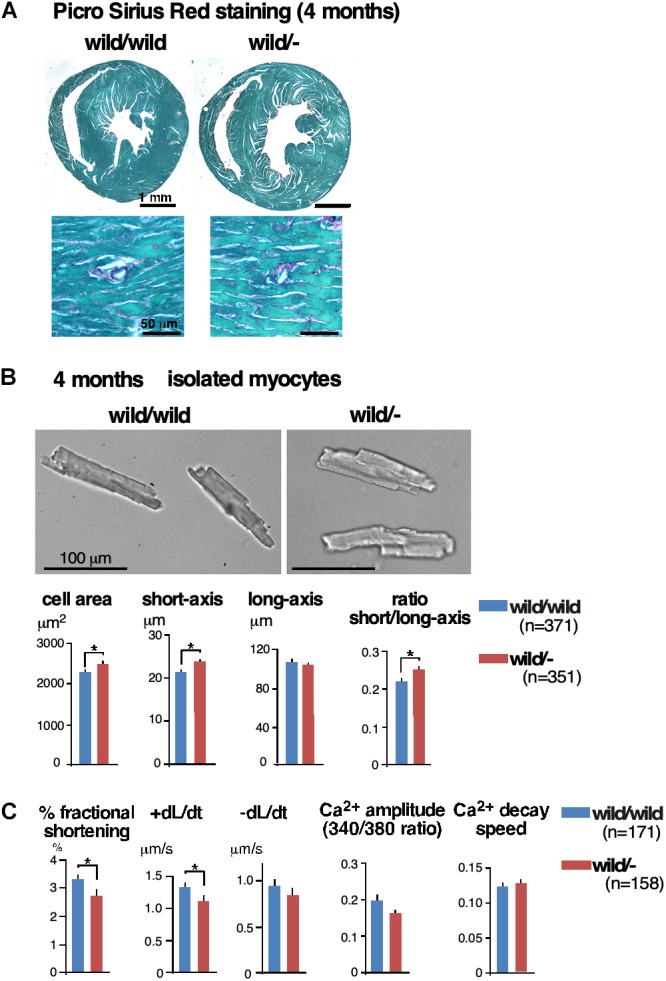
Enlarged cardiomyocytes with reduced contractility and impaired Ca^2+^-handling in heterozygous *Mylk3* knockout mice. **(A)** Representative images of Picro Sirius Red-stained transverse sections of the hearts. **(B)** Representative images of cardiomyocytes, short axis (μm), long axis (μm), cell area (μm^2^) and ratio of short vs. long axis of cardiomyocytes isolated at 4 months of age (*Mylk3*^wild/wild^, *n* = 371; *Mylk3*^wild/-^, *n* = 351 from *n* = 3 mice each). Bars = 100 μm. **(C)** Measurements of cardiac contraction and simultaneous Ca^2+^ transients in isolated cardiomyocytes (*Mylk3*^wild/wild^, *n* = 171; *Mylk3*^wild/-^, *n* = 158 from *n* = 3 mice each). Data are expressed as mean ± SEM. ^∗^*P* < 0.05. +dL/dt (speed of contraction); –dL/dt (speed of relaxation).

Histologically, we did not find interstitial fibrosis, which is often seen in the failed heart, in either of two groups using Picro Sirus Red staining (**Figure 2A**, *n* = 3 each). Of note, we did not see interstitial fibrosis in the homozygous knockout mice despite the higher degree of cardiac dysfunction.

### Cardiomyocyte Enlargement and Reduction in Contractility in Heterozygous *Mylk3* Knockout Mice

Isolated ventricular cardiomyocytes from 4-month-old heterozygous knockout mice were larger in cell surface area accompanied by increased short-axis length and an increased ratio of short axis relative to long axis compared to those isolated from control mice (cell area, 2,300 ± 50 in control vs. 2,500 ± 57 μm^2^ in heterozygous knockout; short axis, 21.5 ± 0.4 vs. 23.9 ± 0.4 μm; short/long axis ratio 0.22 ± 0.1 vs. 0.25 ± 0.01, *n* = 371 and 351, respectively, *P* < 0.05; **Figure 2B**). These cardiomyocytes displayed reductions in contractility and speed of contraction (+dL/dt) without significant changes in amplitude of the intracellular Ca^2+^ transient (%fractional shortening, 3.31 ± 0.17 in control vs. 2.70 ± 0.2% in heterozygous knockout; +dL/dt, 1.33 ± 0.06 vs. 1.11 ± 0.07 μm/sec, *n* = 171 and 158, respectively, *P* < 0.05; **Figure 2C**).

### cMLCK’s mRNA and Protein Expression in Heterozygous *Mylk3* Knockout Mice

**FIGURE 3:**
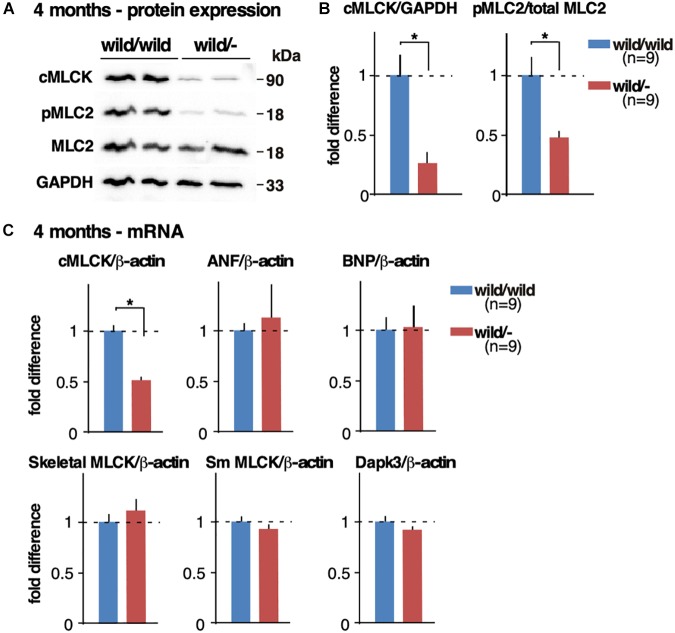
Reduced cMLCK proteins and MLC2 phosphorylation in heterozygous *Mylk3* knockout hearts. **(A)** Representative Western blotting of cMLCK, pMLC2v, total MLC2v and GAPDH using isolated hearts from *Mylk3*^wild/wild^ and *Mylk3*^wild/-^ mice at 4 months of age. The cropped blots are used in the figure. **(B)** Fold difference of cMLCK, pMLC2v and total MLC2v normalized to GAPDH with the value of *Mylk3*^wild/wild^ defined as 1 (*n* = 9 each). **(C)** Real-time RT-PCR demonstrates relative expression of cMLCK, atrial natriuretic factor (ANF), brain natriuretic peptide (BNP), skeletal MLCK, smooth muscle MLCK, and Dapk3 mRNA normalized to β-actin with the value in *Mylk3*^wild/wild^ defined as 1. Data are expressed as mean ± SEM. ^∗^*P* < 0.05. ANF, atrial natriuretic factor; BNP, brain natriuretic peptide; Sm, smooth muscle.

At 4 months of age, cMLCK protein expression was reduced by more than half (approximately by 75%) and MLC2v phosphorylation was reduced by half in heterozygous knockout hearts relative to controls (fold difference cMLCK/GAPDH, 1.00 ± 0.16 in control vs. 0.26 ± 0.09 in heterozygous knockout; fold difference pMLC2/total MLC2, 1.00 ± 0.17 vs. 0.47 ± 0.06, *n* = 9 each, *P* < 0.05; **Figure 3A,B**). Of note, we have previously validated a specificity of phospho-specific MLC2 antibody using 2D-gel followed by Western blotting (Warren et al., 2012).

cMLCK mRNA relative to ß-actin was reduced approximately by half in heterozygous knockout hearts relative to controls (fold difference cMLCK/ß-actin, 1.00 ± 0.05 in control vs. 0.51 ± 0.03 in heterozygous knockout, *n* = 9 each, experiment duplicated and averaged, *P* < 0.05; **Figure 3C**). Expression of mRNA for ANF and BNP relative to ß-actin was not significantly different between the two groups (**Figure 3C**), which is consistent with the lack of increase in heart failure markers present in homozygous sedentary knockout mice (Warren et al., 2012). Despite skeletal and smooth muscle MLCK and Dapk3/ZIPK having been shown to phosphorylate MLC2 (Herring et al., 2000; Davis et al., 2001; Chang et al., 2010), there were no compensatory increases of mRNA of these kinases in heterozygous knockout hearts (**Figure 3C**). This finding was consistent with the homozygous inducible and germline *Mylk3* knockout mice, where cMLCK and MLC2 phosphorylation was markedly reduced or absent (Massengill et al., 2016).

### Reduction of cMLCK Proteins in Heterozygous Knockout Mice

**FIGURE 4:**
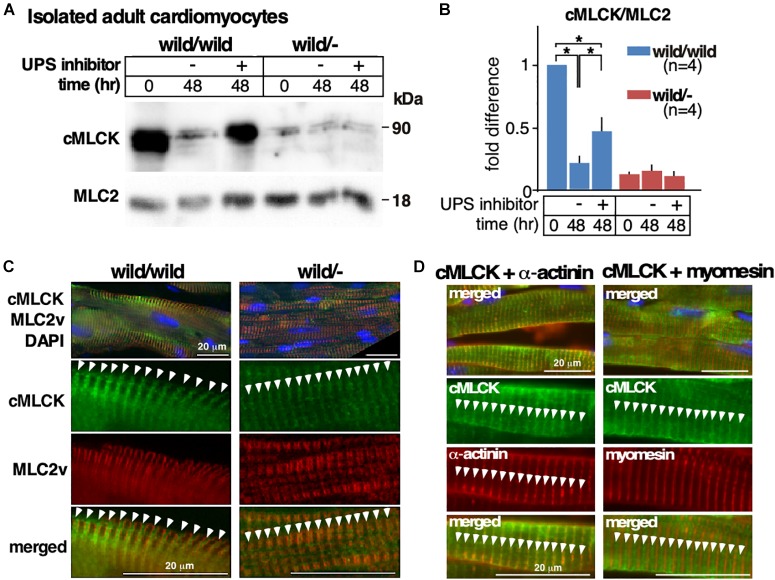
Altered responses to UPS inhibitors and cycloheximide between heterozygous knockout and wild-type adult cardiomyocytes. **(A)** Representative Western blotting of cMLCK and total MLC2v using adult cardiomyocytes isolated from *Mylk3*^wild/wild^ and *Mylk3*^wild/-^ mice at 4 months of age at time 0 and 48 h of incubation with or without UPS inhibitors. The cropped blots are used in the figure. **(B)** Fold difference of cMLCK relative to total MLC2v with the value of *Mylk3*^wild/wild^ at time 0 defined as 1 (*n* = 4 each). **(C)** Representative immunostaining of cMLCK and MLC2v in the heart sections (*n* = 3 each). **(D)** Representative immunostaining of cMLCK and Z-disk protein α-actinin2, and cMLCK and M-line protein myomesin in the heart sections. Data are expressed as mean ± SEM. ^∗^*P* < 0.05. Bars = 20 μm.

We previously showed that the ubiquitin-proteasome system (UPS) is partly responsible for degrading cMLCK proteins in wild-type hearts and neonatal cardiomyocytes (Warren et al., 2012). Using isolated adult cardiomyocytes, we compared the involvement of UPS-dependent protein degradation between heterozygous knockout relative to control. In control cardiomyocytes, unexpectedly, an expression of cMLCK protein was markedly decreased after 48 h of culture (**Figure 4A,B**). However, reduction of cMLCK proteins was partly blocked by UPS inhibitors (combination of lactacystin and MG132) as expected. In contrast, the expression of cMLCK proteins in heterozygous knockout cardiomyocytes was lower compared to those from wild-type mice at time 0, but was not significantly changed within 48 h with or without UPS inhibitors (**Figure 4A,B**). Thus, UPS-dependent protein degradation may not be responsible for the reduction of cMLCK proteins in heterozygous knockout cardiomyocytes.

Despite the marked reduction of cMLCK proteins in heterozygous knockout relative to control hearts, intracellular localization of cMLCK in hearts was similar, showing a striated pattern with additional diffuse staining in the cytoplasm (**Figure 4C**, green). Striated cMLCK staining was not co-localized with MLC2v (**Figure 4C**, red) both in wild-type as well as heterozygous knockout hearts, which is consistent with our previous study (Chan et al., 2008). Co-immunostaining of cMLCK and Z-disk protein α-actinin2 revealed overlapping localization, while co-immunostaining of cMLCK and M-line protein myomesin did not in mouse hearts (**Figure 4D**).

## Discussion

Reduced phosphorylation of MLC2v, a process primarily regulated by cMLCK, has been implicated in human heart disease (Morano, 1999; van der Velden et al., 2003). Recent studies have proposed a novel role for cMLCK in the pathogenesis of DCM following the identification of heterozygous variants in the *MYLK3* gene encoding cMLCK in individuals with disease (Tobita et al., 2017; Hodatsu et al., 2019). To consider the possibility that these heterozygous variants could be loss-of-function mutations (haploinsufficiency), we analyzed heterozygous *Mylk3* knockout mice for the first time.

The young mice at 4 months of age showed mild cardiac dysfunction with increased cardiomyocyte area size and short-axis length relative to the cardiomyocytes from control mice. Expression of cMLCK mRNA was expectedly reduced by almost half, however, protein expression was further reduced in heterozygous knockout relative to wild-type mice. Notably, it is often found that heterozygous knockout mice express more or less than half of the proteins relative to control wild-type mice in other genes (Gineste et al., 2013; Zhou et al., 2015; Zhang et al., 2017). An approximate 75% reduction in cMLCK proteins and a consequent reduction in MLC2 phosphorylation by approximately half may lead to reduced contractility if the observation in human failing hearts is applied to mice. An approximate 50% reduction in MLC2 phosphorylation was found in human failing hearts (Morano, 1999; van der Velden et al., 2003). In normal wild-type mice, two serine residues positioning at 15 and 16 in MLC2v are phosphorylated (Sheikh et al., 2012). When these two serine residues were mutated into alanine in homozygous knock-in mice, these mice developed the DCM phenotype accompanied with loss of MLC2v phosphorylation (Sheikh et al., 2012). Whether heterozygous knock-in mice develop heart failure remains to be studied, which will help us to understand whether haploinsufficiency of the *Mylk3* gene or a reduction in MLC2 phosphorylation is the primary or secondary trigger of cardiac dysfunction observed in heterozygous *Mylk3* knockout mice.

The phenotype demonstrated in heterozygous *Mylk3* knockout mice is milder than the reported phenotype seen in humans with heterozygous variants in the *MYLK3* gene. There are several possible explanations for the phenotypic difference between heterozygous variants in human and heterozygous knockout in mice. It could be due to species effects, in other words mouse as opposed to man (Hacking, 2008). A second possibility is that the effect of human variants may differ from the effect of the haploinsufficiency resulting from heterozygous knockout. cMLCK proteins are composed of roughly three different domains: the amino terminal domain unique to the cardiac isoform without homologies to other MLCKs such as smooth muscle or skeletal types, followed by the conserved catalytic and regulatory domains (Seguchi et al., 2007; Chan et al., 2008). To our knowledge, three different mutations in *MYLK3* gene have been identified (Tobita et al., 2017; Hodatsu et al., 2019). Two mutations are predicted to generate a shorter protein with disruption of the catalytic and regulatory domains. Another mutation at the stop codon is predicted to generate a longer protein with an additional 19 amino acids at the carboxyl-terminus. It might be anticipated that for such diverse effects, such as haploinsufficiency compared with dominant-negative, there would be associated variation in clinical phenotypes (Kasahara and Benson, 2004). A third possibility is that patients with heterozygous mutations in *MYLK3* gene may carry additional genetic mutations in other genes that may contribute to the phenotypic presentation. In fact, an additional nonsense mutation in a filamin C (FLNC) gene was found in some of the family members exhibiting early onset of DCM (Tobita et al., 2017), and genetic linkages between the FLNC gene and DCM have been reported (Ortiz-Genga et al., 2016; Reinstein et al., 2016).

Despite that one allele in the human *MYLK3* gene being intact in these patients, cMLCK protein expression in patients’ hearts was reported to be markedly reduced compared to control (Tobita et al., 2017). In heterozygous *Mylk3* knockout hearts, we also observed a marked reduction of cMLCK proteins relative to the wild-type mice. We attempted to compare the differences in UPS-dependent protein degradation between heterozygous knockout and controls using isolated adult cardiomyocytes. In the wild-type cardiomyocytes cMLCK proteins were markedly downregulated within 48 h of culture, and this was partly blocked by UPS inhibitors. On the other hand, expression of cMLCK in heterozygous knockout cardiomyocytes was lower than wild-type cardiomyocytes shortly after isolation from hearts, but was not significantly changed within 48 h of culture regardless of UPS inhibitor presence. These results indicate that expression of cMLCK protein in hearts and isolated cardiomyocytes is regulated by its environment, namely, the dynamic environment of contractile hearts with over 600 beats per minute *in vivo* versus the static environment of isolated cardiomyocytes. In addition, there were distinct differences between wild-type and heterozygous knockout cardiomyocytes regarding the abundance of cMLCK proteins during 48 h of culture and responses to UPS inhibitors; however, our hypothesis regarding the increase of UPS-dependent protein degradation in heterozygous knockout cardiomyocytes relative to control was not supported.

The intracellular localization of the cMLCK protein was similar between control and heterozygous cMLCK knockout mice, showing a striated pattern co-localized not with MLC2v, but with Z-disk protein alpha-actinin, with additional diffuse staining in the cytoplasm. Studies into whether this intracellular localization has additional roles in cMLCK are currently underway.

In summary, we report that the heterozygous loss-of-function mutation of *Mylk3* in mice shows a mild reduction in cardiac contractility and a reduction in proteins by approximately 75% relative to the control wild-type mice. These phenotypes partly represent DCM in humans with the heterozygous *MYLK3* mutation, but are milder. This animal model has the potential to shed light on understanding the mechanisms of low cMLCK protein expression in human DCM patients with heterozygous *MYLK3* mutations.

## Ethics Statement

This study was carried out in accordance with the recommendations of the NIH guidelines. The protocol was approved by University of Florida Institutional Animal Care and Use Committee.

## Author Contributions

All authors designed and performed the experiments. CT and HK prepared the manuscript.

## Conflict of Interest Statement

The authors declare that the research was conducted in the absence of any commercial or financial relationships that could be construed as a potential conflict of interest.
