# Supplementary material for: Heterozygous Mylk3 Knockout Mice Partially Recapitulate Human DCM With Heterozygous MYLK3 Mutations
Source: Front Physiol. 2019 Jun 6;10:696. doi: 10.3389/fphys.2019.00696 (PMC6563786; doi:10.3389/fphys.2019.00696)

Supplemental Figure 1. Full-length blots used for Figure 3A.

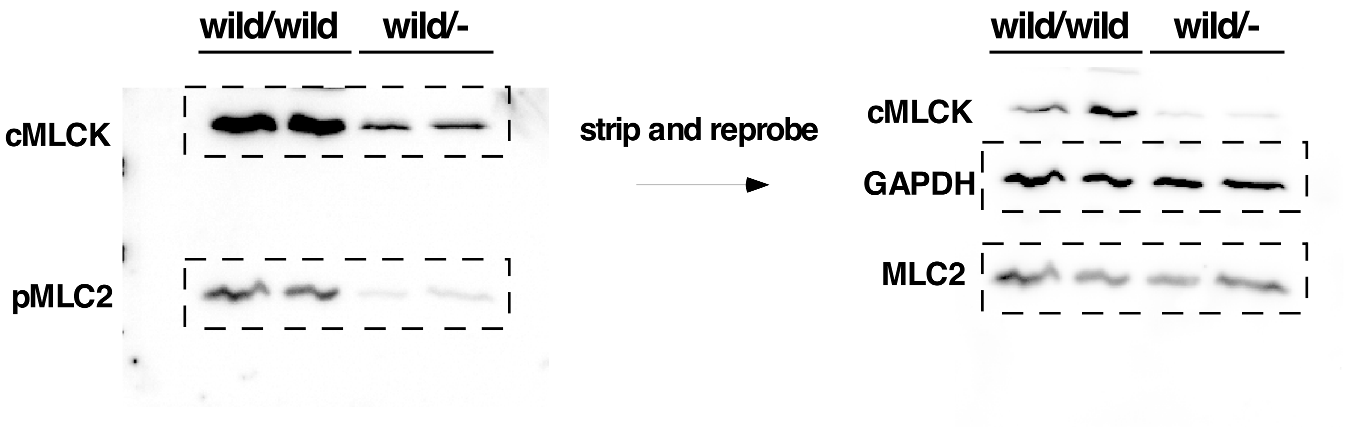

Supplemental Figure 2. Full-length blots used for Figure 4A.

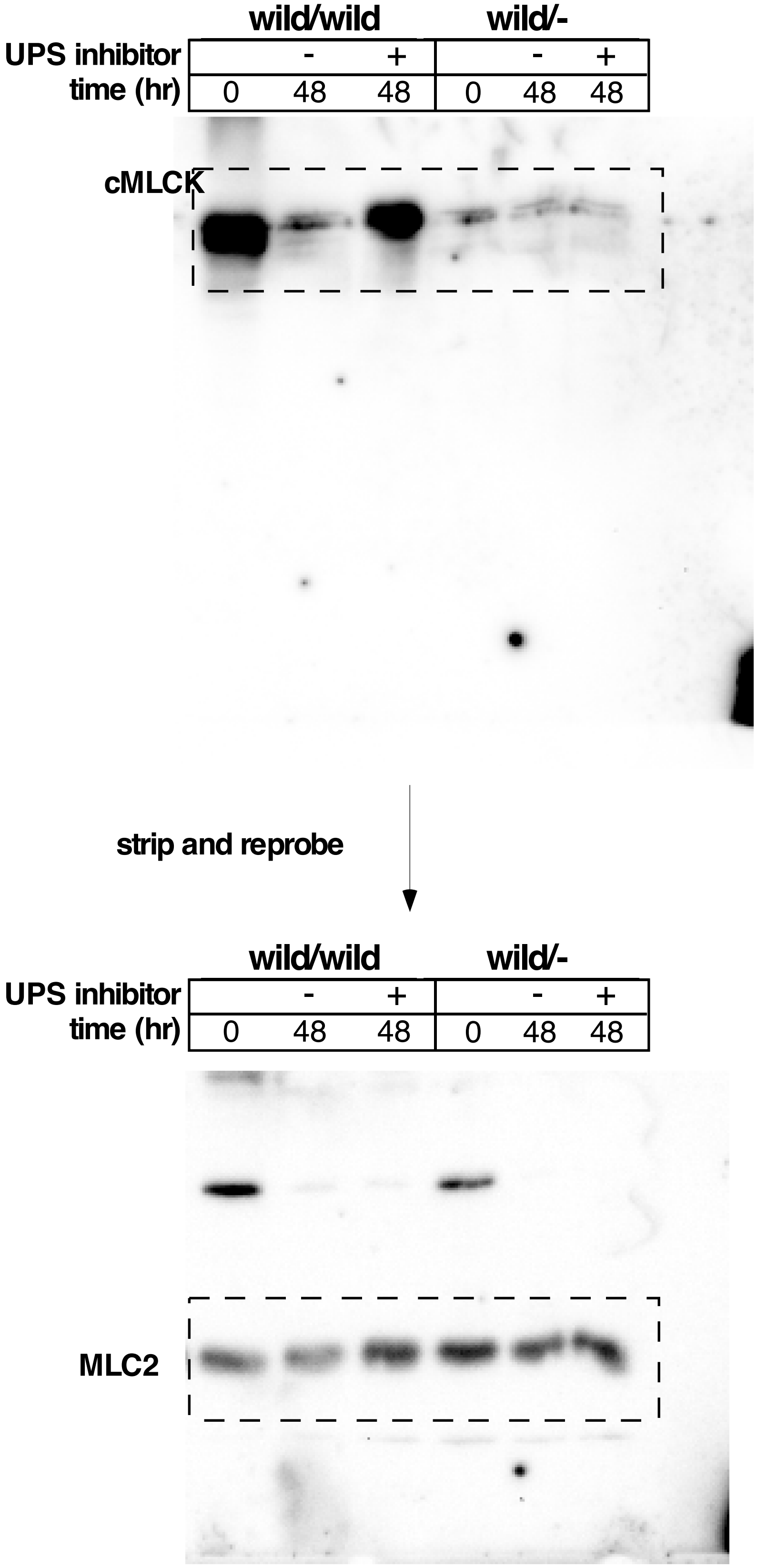

Supplement: Supplementary file 1 [file Presentation_1.PDF]
